# Progress in Electrochemical Immunosensors with Alkaline Phosphatase as the Signal Label

**DOI:** 10.3390/bios13090855

**Published:** 2023-08-29

**Authors:** Changdong Chen, Ming La, Xinyao Yi, Mengjie Huang, Ning Xia, Yanbiao Zhou

**Affiliations:** 1 College of Chemical and Environmental Engineering, Pingdingshan University, Pingdingshan 476000, China; 2 College of Chemistry and Chemical Engineering, Central South University, Changsha 410083, China; 3 College of Chemistry and Chemical Engineering, Anyang Normal University, Anyang 455000, China

**Keywords:** alkaline phosphatase, electrochemical immunosensors, signal amplification, redox cycling, nanoparticles

## Abstract

Electrochemical immunosensors have shown great potential in clinical diagnosis, food safety, environmental protection, and other fields. The feasible and innovative combination of enzyme catalysis and other signal-amplified elements has yielded exciting progress in the development of electrochemical immunosensors. Alkaline phosphatase (ALP) is one of the most popularly used enzyme reporters in bioassays. It has been widely utilized to design electrochemical immunosensors owing to its significant advantages (e.g., high catalytic activity, high turnover number, and excellent substrate specificity). In this work, we summarized the achievements of electrochemical immunosensors with ALP as the signal reporter. We mainly focused on detection principles and signal amplification strategies and briefly discussed the challenges regarding how to further improve the performance of ALP-based immunoassays.

## 1. Introduction

Electrochemical immunosensors are considered as the most widely used detection techniques in the fields of food safety, disease diagnosis, and environmental monitoring because of their intrinsic merits of high selectivity and sensitivity, rapid response, and ease of miniaturization [1,2,3,4,5,6]. In order to improve the sensitivity, particular attention is paid to exploit a series of effective signal amplification strategies for the detection of low-abundance targets, including enzyme catalysis, DNA assembly, and nanomaterials [7,8,9,10]. For example, when DNA–antibody conjugates are introduced into immunosensors, the previously reported amplification techniques for DNA detection can be employed for the ultrasensitive detection of antigens, such as the hybridization chain reaction (HCR), strand displacement amplification (SDA), and rolling circle amplification (RCA) [11,12]. Magnetic nanoparticles are widely used in electrochemical immunoassays due to their remarkable merits in the separation and pre-concentration of targets from complex biological samples [13,14,15]. Enzyme catalysis can be perfectly integrated with these amplification techniques to improve the sensitivity of electrochemical immunosensors, thus favoring their applications in bioanalytical fields [16,17,18].

Nowadays, enzymes such as horseradish peroxidase (HRP), alkaline phosphatase (ALP), glucose oxidase, and tyrosinase are successfully utilized as signal labels to amplify electrochemical signals [19,20]. Among them, HRP and ALP are two of the most popularly used enzyme reporters [21]. Nonetheless, the application of HRP may be affected by several inherent problems, such as a non-specific staining response, activity inhibition by Cu^+^ ions, various microorganisms and antibiotics, and high background from the electrochemical reduction of the H_2_O_2_ substrate. In contrast, ALP has attracted considerable attention as a reporter enzyme used for signal amplification due to its excellent advantages of high catalytic activity, a high turnover number (1000-fold higher than that of HRP), and broad substrate specificity [22]. Despite all this, the sensitivity of ALP-based electrochemical immunosensors is still relatively limited. Aiming to successfully achieve high sensitivity and a low detection limit (LOD), other strategies or devices can be elaborately combined with ALP to boost performance [23]. For example, nanolabels modified with a large number of ALP molecules and cascade reactions between ALP and nanocatalysts/nanozymes are successfully used to construct highly sensitive electrochemical immunosensors. Immunoreaction-triggered DNA nanostructures can capture plenty of ALP molecules via avidin–biotin interactions. Several reviews introduce the role of ALP in electrochemical bioassays [24,25,26]. However, there are few systematic reviews that focus on the advancements of electrochemical immunosensors with ALP as the signal label. In this work, we aim to comprehensively summarize the development of ALP-based electrochemical immunosensors from three sections according to the types of signal outputs, including the electrochemical, photoelectrochemical (PEC), and electrochemiluminescent (ECL) methods. In each section, the works are carefully categorized based on the differences of the catalytic reactions and signal outputs. The main problems and future perspectives are also discussed. Due to space limitations and incomprehensive bibliographic retrieval, we apologize for the omission of some interesting and important works.

## 2. Electrochemical Methods

Although antigen–antibody interactions can be monitored by label-free electrochemical methods, the small physicochemical changes derived from the binding of an antigen to an antibody lead to a weak electrochemical signal [27]. For the sensitive detection of analytes at low concentrations, enzymes are frequently used as labels to modify an antibody or an antigen for converting binding events to detectable electrochemical signals. Among the kinds of reporting enzymes, ALP is one of the most popularly used signal labels in electrochemical immunoassays because of its high turnover number, low cost, and high stability. The detection principles of ALP-based electrochemical immunosensors are mainly dependent upon enzymatic products that are linearly proportional to the target concentration [28]. The simplest detection principle is to directly quantify the electroactive products generated by ALP catalysis through different electrochemical techniques. By taking advantages of the characteristics of ALP substrates and products, different signal amplification strategies, including redox cycling and product-triggered in situ metallization, are introduced into detection systems to amplify the signals [29].Thus, ALP-based electrochemical immunoassays are widely constructed to determine various targets, including nucleic acids, proteins, bacteria, viruses, biological toxins, andother microorganisms in food matrices [30,31]. In addition, such immunoassays are also developed to determine the pesticides and antibiotics in the environment and agriculture [32,33,34]. The detailed signal amplification strategies and principles are reviewed in the following subsections, and the analytical performance of typical examples is shown in Table 1.

### 2.1. Direct Detection of ALP-Catalyzed Products

**Figure 1:**
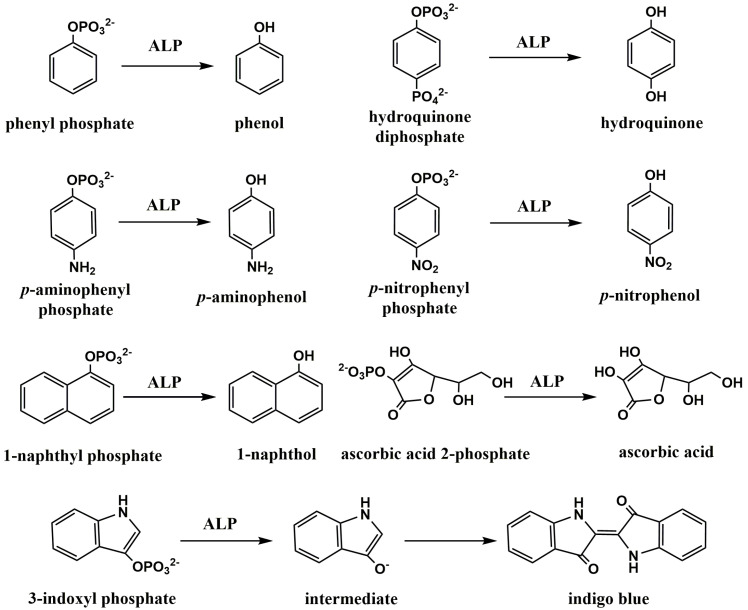
The chemical structure of several typical ALP substrates and products.

ALP-based immunosensors can be constructed on the electrode surface in which the enzymatic products are directly electrochemically reduced or oxidized. The electrochemical response is related to the antigen concentration, and the sensitivity is dependent on the catalytic ability of ALP for substrate hydrolysis. To obtain a high signal-to-background ratio, ALP substrate should be electrochemically inactive within the scanning potential window [35]. To date, a lot of substrate/product pairs for ALP-based electrochemical immunoassays have been exploited with the structures shown in Figure 1. Initially, phenyl phosphate was used as the substrate to construct electrochemical immunosensors for the detection of digoxin and progesterone [36,37]. However, the high oxidative potential of phenol led to a high background signal, and the electropolymerization of phenol radicals fouled the electrode surface, resulting in a loss of sensing performance.

**Figure 2:**
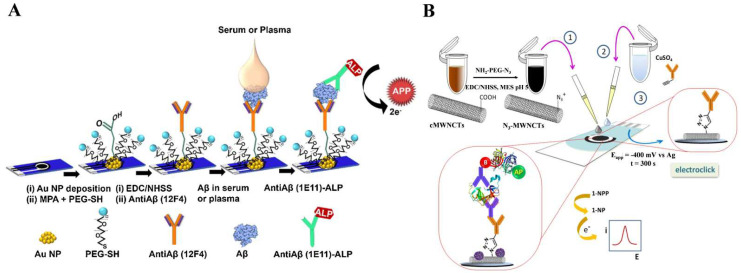
(**A**) Schematic illustration of the electrochemical immunoassay for detection of Aβ peptide using PAPP as the ALP substrate [102]. Copyright 2017 Elsevier. (**B**) Schematic illustration of the preparation process of the ALP-based electrochemical immunosensor for detection of IL-1β cytokine [103]. Copyright 2020 Elsevier.

Aiming to reduce the oxidation potential of enzyme products and avoid electrode fouling, a wide variety of alternative substrate/product pairs were developed for ALP-based electrochemical immunosensors, including *p*-aminophenyl phosphate (PAPP)/*p*-aminophenol (PAP) [38,39,40,41,42,43,44,45,46,47,48,49,50,51,52,53], hydroquinone diphosphate(HQDP)/hydroquinone (HQ) [54,55,56,57,58], *p*-nitrophenylphosphate (PNPP)/*p*-nitrophenol (PNP) [59,60,61,62], α-naphthyl phosphate (NPP)/α-naphthol [63,64,65,66,67,68,69,70,71,72,73,74,75,76,77,78,79,80,81,82,83,84], 3-indoxyl phosphate (3-IP)/indigo blue [85,86,87,88,89], ascorbic acid 2-phosphate (AAP)/ascorbic acid (AA) [90,91,92,93,94], and disodium phenyl phosphate (DPP)/phenol [95,96,97,98]. For instance, Preechaworapun et al. reported an ALP-based immunosensor for the amperometric detection of mouse IgG using AAP as the substrate [99]. PAPP was utilized as the substrate for the electrochemical determination of cancer cells, immunoglobulin E, and the amyloid-beta 1–42 (Aβ) peptide [100,101,102]. As profiled in Figure 2A, gold nanoparticles (AuNPs)-decorated screen-printed carbon electrode (SPCE) was modified with mercaptopropionic acid (MPA) and thiol-functionalized polyethylene glycol (PEG-SH). The antibody toward Aβ, antiAβ(12F4), was immobilized on the surface of SPCE through *N*-(3-dimethylaminopropyl)-*N*’-ethylcarbodiimide hydrochloride/*N*-hydroxysulfosuccinimide (EDC/NHSS)-mediated coupling chemistry. The peptides of Aβ in biological samples were captured and then labeled with ALP-conjugated antiAβ(1E11). The ALP-catalyzed hydrolysis of PAPP to PAP was electrochemically monitored. In addition, Guerrero et al. developed an electrochemical immunosensor for the detection of IL-1β cytokine through electro-click chemistry [103]. As depicted in Figure 2B, ethynylated IgG was immobilized on azide-functionalized multi-walled carbon nanotubes (MWCNTs)-modified SPCE through the Cu(I)-catalyzed cycloaddition reaction for the capture of the IL-1β cytokine and ALP-linked detection antibody. After the sandwich immunoreaction, ALP catalyzed the hydrolysis of NPP to NP that could be determined by differential pulse voltammetry (DPV).

**Figure 3:**
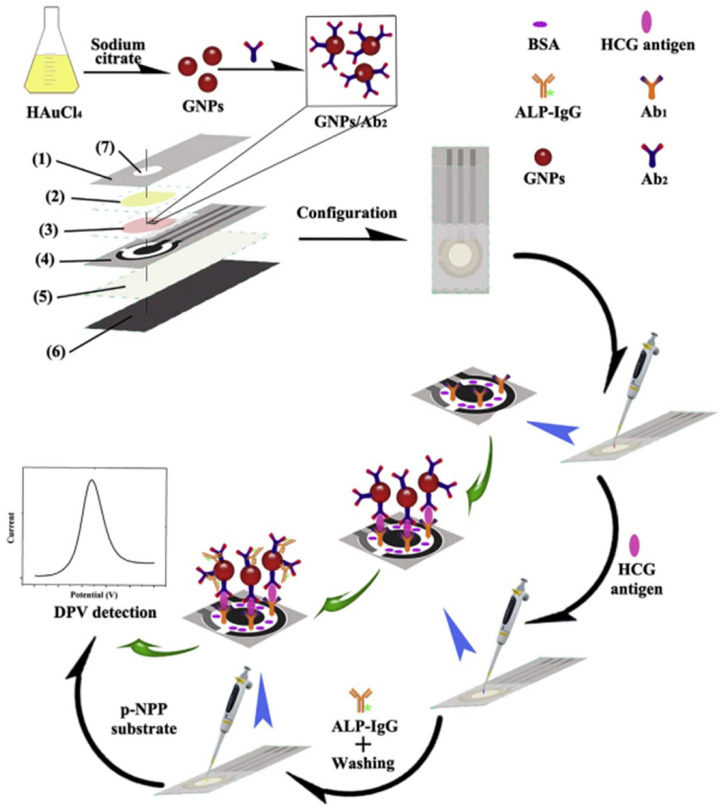
Schematic illustration of assembly processes and assay procedure of the μPAD-based electrochemical immunosensor. (1) ABS top cover, (2) cellulose paper-based sample pad, (3) glass fiber-based conjugate pad, (4) paper-based SPEs, (5) blotting paper (6) single-sided adhesive backing layer, and (7) sample injection hole [109]. Copyright 2017 Elsevier.

In order to further improve the detection sensitivity, multi-enzyme strategies were proposed with a variety of nanomaterials as the carriers to load plenty of enzymes [104,105,106,107,108]. For instance, Cao et al. fabricated a microfluidic paper-based device (μPAD) for the electrochemical immunoassay of human chorionic gonadotropin (HCG) using Ab_2_-AuNPs as the recognition elements [109]. As illustrated in Figure 3, Ab_2_-AuNPs were used to label the captured HCG, and many ALP-linked secondary antibodies (ALP-IgG) were specifically adsorbed on Ab_2_-AuNPs. DPV was used to measure the electrochemical signal from the ALP-catalyzed conversion of PNPP to PNP. Hou et al. reported an electrochemical immunosensor for the detection of tumor necrosis factor α (TNF-α) based on ALP-catalyzed generation of AA and hydrogel prepared from ferrocene (Fc)-modified amino acids [110]. In this work, the hydrogel formed from the self-assembly of Fc-modified phenylalanine showed high redox activity due to the large number of Fc moieties. However, the ALP-catalyzed generation of AA led to the reduction of the Fc moieties in hydrogel, which was accompanied by a decrease in the redox current.

### 2.2. ALP Catalysis plus Redox Cycling

Redox cycling involves the repetitive generation of signal molecules in the presence of additional reducing or oxidative species [111]. In this process, a small number of signal molecules can produce a significantly enhanced electrochemical signal. Nowadays, electrochemical immunosensors based on the signal amplification of enzymes plus electrochemical–chemical (EC) or electrochemical–chemical–chemical (ECC) redox cycling arouse wide interest due to their excellent sensitivity and selectivity [112,113,114,115,116,117].

#### 2.2.1. EC Redox Cycling

**Figure 4:**
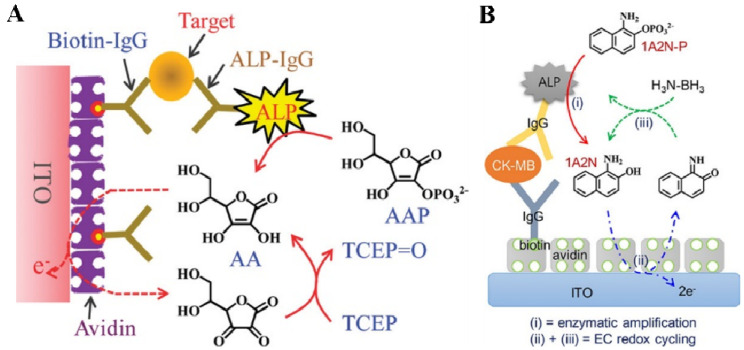
(**A**) Schematic illustration of the electrochemical immunosensor for troponin I detection using the generation of AA by ALP and the redox cycling of AA by TCEP [118]. Copyright 2011 American Chemical Society. (**B**) Schematic illustration of the electrochemical immunosensor for the detection of CK-MB using (i) enzymatic amplification and (ii) + (iii) EC redox cycling [119]. Copyright 2017 American Chemical Society.

In typical ALP plus EC redox cycling, the substrate should be electrochemically inactive, and the product can be electrochemically oxidized on the electrode surface at a relatively low potential with a high reaction rate. Then, the oxidized product could be immediately reduced by a reducing agent for the next electrochemical oxidation. Thus, a few enzymatic products can be repeatedly regenerated through chemical redox cycling reactions by excessive reducing agents, ultimately producing a strongly amplified electrochemical signal. Meanwhile, the reducing agents can protect the enzymatic products from oxidation in the ambient air. In order to minimize the background current, indium–tin oxide (ITO) electrodes are widely used in EC redox-cycling-based immunoassays because of their low electrocatalytic activity toward reducing agents. Akanda et al. developed an ALP and EC redox-cycling-based immunosensor for the detection of troponin I (Figure 4A) [118]. The performance of AAP was compared with that of other ALP substrates (e.g., PAPP, NPP, and 4-amino-1-naphthyl phosphate). It was found that the AAP/AA (substrate/product) pair was better than the others in terms of the formal potential and the electro-oxidation rate. The ITO electrode without immobilization of the electrocatalyst or electron mediator exhibited a good voltammetric behavior for the fast electro-oxidation of AA. Meanwhile, tris(2-carboxyethyl)phosphine (TCEP) allowed for fast redox cycling with a very low anodic current at the electrode. In the presence of TCEP, the enzymatic product (AA) produced from the ALP-catalyzed hydrolysis of AAP triggered the redox cycling reaction.

The electrochemical oxidation of diaromatic substances is faster than that of monoaromatic reagents with lower electrocatalytic ability. However, such substances are easily oxidized by dissolved oxygen, limiting their applications in EC redox cycling. To address this problem, Seo et al. developed an electrochemical immunosensor for the detection of creatine kinase-MB (CK-MB) using 1-amino-2-naphthyl phosphate (1A2N-P) and H_3_N-BH_3_ as the ALP substrate and reducing reagent for EC redox cycling, respectively (Figure 4B) [119]. In this study, ALP catalyzed the hydrolysis of stable 1A2N-P to 1-amino-2-naphthol (1A2N). The oxygen-caused oxidation and polymerization of 1A2N was prevented by excessive H_3_N-BH_3_. Meanwhile, the EC redox cycling between 1A2N and H_3_N-BH_3_ produced a strong electrochemical signal for CK-MB determination.

**Figure 5:**
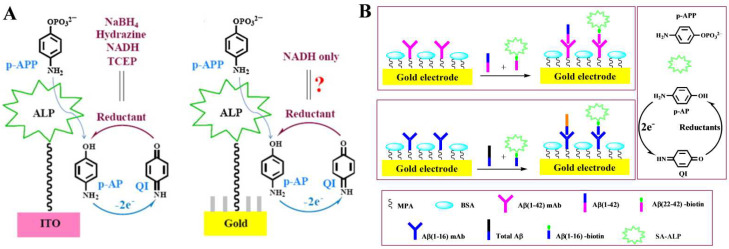
(**A**) Schematic illustration of the process of PAP production and its electrocatalytic reaction by reductants on ITO and gold electrode [120]. Copyright 2013 Elsevier. (**B**) Schematic illustration of the detection of Aβ(1–42) and total Aβ using PAP-mediated redox cycling by chemical reductants [122]. Copyright 2014 Elsevier.

The low activity of the ITO electrode may result in a low electro-oxidation rate for enzymatic products, leading to a weak signal. Therefore, it is important to select appropriate ALP products, reducing agents, and sensing electrodes. Usually, a gold electrode with electrocatalytic ability is not suitable for redox cycling because the reducing agent may exhibit a relatively low potential at the electrode surface, causing a high background current. Challenges remain to find appropriate reducing agents for redox cycling with a low background current. One of our groups systematically evaluated the performance of biosensors with different reducing agents, including NaBH_4_, hydrazine, TCEP, nicotinamide adenine dinucleotide (NADH), Na_2_SO_3_, and cysteamine on analkanethiol self-assembled monolayers (SAMs)-modified gold electrode (Figure 5A) [120]. The result suggested that NADH, TCEP, and cysteamine were suitable for PAP-mediated EC redox cycling because of their low background current on the SAMs-modified gold electrode [121]. Based on PAP-mediated EC redox cycling, Liu’s group developed a competitive electrochemical immunosensor for the detection of Aβ(1–42) and total Aβ peptides [122]. As displayed in Figure 5B, Aβ(1–42) could compete with biotin-Aβ(22–42) to bind Aβ(1–42)mAb on the electrode through the immunoreaction. Similarly, total Aβ peptides could compete with biotin-Aβ(1–16) to bind Aβ(1–16) mAb on the electrode. After the competitive immunoreaction, the streptavidin (SA)-conjugated ALP (SA-ALP) captured by the sensor electrode catalyzed the hydrolysis of PAPP to PAP, initiating the EC redox cycling in the presence of TCEP to generate an amplified anodic current. In addition, to avoid the potential impact of redox species in samples, an immunomagnetic pre-concentration was combined with ALP and EC redox cycling for the immunoassay of Salmonella [123]. After magnetic separation, the enzymatic product (AA) triggered the EC redox cycling in the presence of TCEP. The immunoassay showed a detection limit of 6.0 × 10^2^ CFU/mL in agricultural water.

The electrochemical detection of enzymatic products by direct oxidation at the sensor electrode exhibits slow electron-transfer kinetics and high surface fouling from oxidation products, thus resulting in poor sensitivity, selectivity, and reproducibility. In order to avoid or minimize this effect, redox mediators or nanocatalysts can be introduced to accelerate the oxidation of enzymatic products, in the form of surface-confined layers or the electrolyte solution phase [124]. Actually, the redox mediators or the nanocatalysts-accelerated oxidation of ALP products also involve the redox cycling process. Unlike the abovementioned works, the mediator is electro-oxidized on the electrode surface, and its oxidized form is reduced back to the reduced form by the abundant ALP products accumulated in the solution. Then, the regenerated mediator in the reduced form is electrochemically oxidized again, eventually producing an amplified signal. Notably, the mediator should exhibit faster electron-transfer kinetics and lower formal reversible potential than the enzymatic products, as well as good stability in both oxidized and reduced forms. It was shown that Fc and its derivatives can electrochemically catalyze the oxidation of AA, thereby amplifying the current [125,126]. Zhong et al. developed an electrochemical immunosensor for human apurinic/apyrimidinic endonuclease 1 (APE-1) detection using ALP and Fc-tagged Ab_2_-modified AuNPs-decorated graphene nanosheets as the signal labels [127]. In this work, the Fc/Fc^+^ couple effectively catalyzed the electrochemical oxidation of the enzymatic product (AA), further increasing the current response.

**Figure 6:**
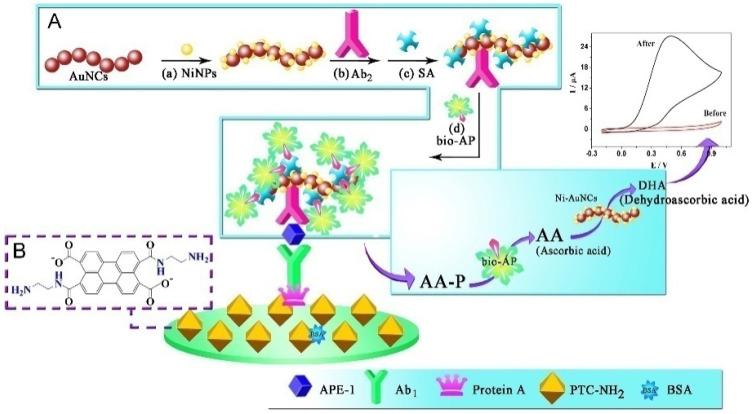
Schematic illustration of the prepared immunosensor for APE-1 detection and the triple signal amplification mechanism: (**A**) the stepwise bio-AP/SA/Ab_2_/Ni–AuNCs bioconjugates fabrication process: (**a**) absorption of NiNPs, (**b**) Ab_2_ loading, (**c**) blocking with SA, and (**d**) binding bio-AP; (**B**) the molecular structure of PTC-NH_2_ [131]. Copyright 2013 Elsevier.

Like the mediator, nanomaterials can also serve as electrocatalysts to accelerate the electrochemical oxidation of enzymatic products, in the form of electrode substrates or signal labels [7]. For this consideration, ALP-based electrochemical immunoassays were extensively developed by coupling the ALP-catalyzed in situ generation of enzymatic products with nanomaterials-assisted electrocatalysis [128,129]. For instance, Hayat demonstrated that nanoceria particles could catalyze the oxidation of enzymatic product 1-naphthol [130]. Han et al. proposed an ultrasensitive ALP-based electrochemical approach for the detection of APE-1 via the triple signal amplification strategy [131]. As shown in Figure 6, the organic compound PTC-NH_2_ was synthesized by 3,4,9,10-perylene tetracarboxylic dianhydride and ethylenediamine. A glass carbon electrode (GCE) was modified with PTC-NH_2_ for the covalent immobilization of protein A, which could lower the background current signal. This step was followed by incubation with bovine serum albumin (BSA) solution to block the surface. ALP and Ab_2_ were co-immobilized on the nickel hexacyanoferrates nanoparticle-decorated Au nanochains (Ni–AuNCs) as multienzyme labels, achieving the first signal amplification. After the immunoreaction, the biocatalysis of ALP toward the conversion of AAP to AA achieved the second signal amplification. Concomitantly, the third signal amplification was achieved with the EC redox cycling process among AA, Ni–AuNCs, and the electrode. Based on triple signal amplification, a significantly enhanced electrochemical signal was obtained for APE-1 detection.

#### 2.2.2. ECC Redox Cycling

**Figure 7:**
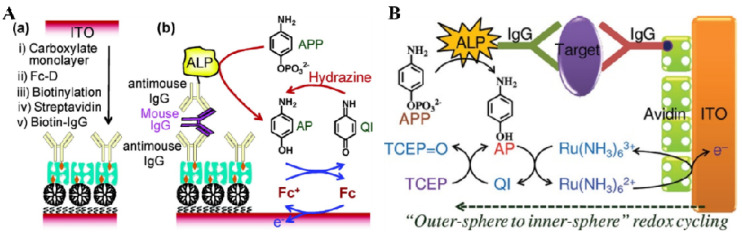
(**A**) Schematic representation of an electrochemical immunosensor for the detection of mouse IgG based on PAP-mediated ECC redox cycling using hydrazine as the reductant. (**a**) Schematic representation of the preparation of an immunosensing layer. (**b**) Schematic view of electrochemical detection for mouse IgG [133]. Copyright 2007 American Chemical Society. (**B**) Schematic representation of “outer-sphere to inner-sphere” redox cycling for ultrasensitive immunosensors [135]. Copyright 2012 American Chemical Society.

ECC redox cycling can be achieved in the presence of an oxidant and a reducing reagent. Usually, the oxidant serves as an electron mediator to catalyze the electrochemical oxidation of ALP products. The generated oxidation products are reduced by excess reducing reagents and then electrochemically oxidized again with the aid of the oxidant mediator. In ECC redox cycling, a few ALP products can greatly promote the current signal. The electrocatalytic activity of the sensor electrode toward the reducing reagent used in the ECC redox cycling may influence the background current and the signal-to-noise ratio. Highly electrocatalytic electrode can lead to a strong background current due to the oxidation of the reducing reagent at a low potential. Although a partially ferrocene-functionalized dendrimer (Fc-D)-modified ITO was employed to design a ECC redox cycling system with NaBH_4_ as the reducing reagent, a gold nanoparticle as the nanocatalyst, and PNP as the substrate [132], the background current for the reduction of NaBH_4_ at the modified ITO is still high. Thus, it is necessary to explore electrochemically inactive and more stable reducing agents for ECC redox cycling. Das et al. developed an electrochemical immunosensor for the detection of mouse IgG based on PAP-mediated ECC redox cycling using hydrazine as the reducing agent (Figure 7A) [133]. In this study, hydrazine was used as a reducing agent for ECC redox cycling because it was electrochemically inactive and exhibited slow electro-oxidation kinetics on the Fc-D-modified ITO electrode. After the formation of a sandwich immunecomplex on the ITO electrode, PAPP was converted to PAP under the catalysis of ALP for a certain incubation time, accumulating a large number of electroactive PAP species. The generated PAP was electrochemically oxidized to *p*-quinoneimine (PQI) under the electrocatalysis of the Fc moieties in Fc-D as the redox mediator. Then, the oxidized PQI was reduced back to PAP by excess hydrazine, and the resulting PAP was again electrochemically oxidized to PQI. Through repeated electrocatalytic reactions, the oxidation current of PAP was dramatically increased, and the sensitivity of the ALP-based immunosensor was significantly improved.

After that, more efforts were put into the selection of suitable ECC systems composed of a reducing reagent, an enzymatic product, a redox mediator, and a sensor electrode. For example, Kwon et al. proposed an ECC redox cycling system with NADH as the reductant [134]. In this study, a gold electrode was modified with the SAMs of long thiol molecules to reduce the background current and was further modified with Fc-D to promote the oxidation of p-AP. NADH showed a slow electrochemical oxidation rate at the electrode and a fast chemical reaction with PAP during the redox cycling. Akanda et al. reported an ECC redox cycling system for an ultrasensitive immunoassay of cardiac troponin I (cTnI) [135]. As shown in Figure 7B, outer-sphere-reaction (OSR)-philic Ru(NH_3_)_6_^3+^, innersphere-reaction (ISR)-philic TCEP, and an OSR- and ISR-philic QI/AP couple were used as the oxidant, reductant, and ALP substrate/product to design an “outer-sphere to inner-sphere” redox cycling system. The QI/AP couple exhibited fast redox reactions with both Ru(NH_3_)_6_^3+^ and TCEP. A highly OSR-philic ITO electrode minimized the unwanted electrochemical reaction with TCEP. The immunosensor showed a detection limit of 10 fg/mL for the detection of troponin I in serum.

**Figure 8:**
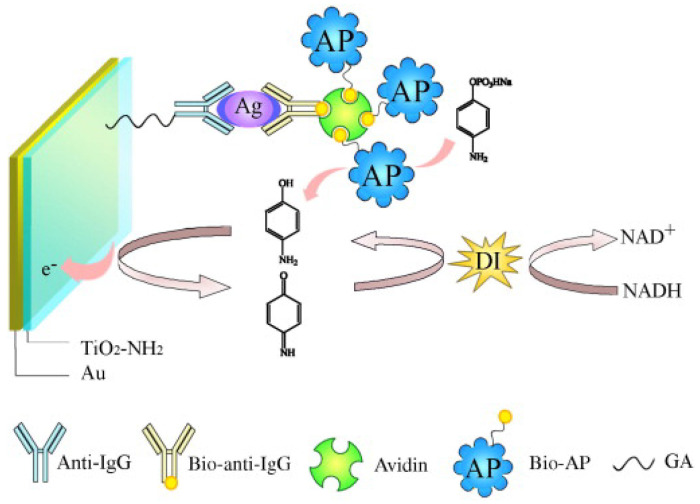
Schematic illustration of assembly process with the enzyme bioaffinity immunosensor for human IgG detection based on bienzyme substrate recycling for amplification [138]. Copyright 2010 Elsevier.

In ALP-mediated enzymatic–enzymatic (EE) redox cycling, appropriate oxidoreductases, such as tyrosinase and diaphorase (DI), are required to regenerate ALP products with the consumption of enzyme substrates [136,137]. For example, Yuan et al. developed an ALP-mediated electrochemical immunosensor for the determination of human IgG based on bienzyme redox cycling [138]. As displayed in Figure 8, under the catalysis of ALP, electroactive PAP was produced from the inactive substrate of PAPP and was concomitantly oxidized at the electrode surface to *p*-quinoneimine (PQI). PQI was then reduced to PAP by DI, leading to the repeated generation of PQI. The oxidized state of DI changed to its native state by the substrate of NADH. Under ALP/DI EE redox cycling, the immunosensor exhibited a wide linear range from 1 × 10^−14^ to 1 × 10^−5^ g/mL with a detection limit of 3.5 × 10^−15^ g/mL.

### 2.3. ALP-Catalyzed Metal Deposition

The enzymatic control of metal precipitation is an effective approach to decrease the background signal in contrast to conventional AuNPs-catalyzed silver electrodeposition. ALP-catalyzed reductive products, such as indoxyl intermediate, PAP, and AA, can reduce Ag^+^ ions to Ag deposited on the electrode with a low background. The amount of Ag deposition can be increased by accumulating the enzymatic products over a long period of incubation, and the oxidation peak can be monitored by anodic stripping voltammetry (ASV). A well-defined ASV peak allowed for the electrochemical immunoassays of various targets with ALP-linked detection antibodies [139,140,141,142]. For example, Chen et al. developed an electrochemical immunosensor for human IgG detection based on ALP-catalyzed Ag deposition [143]. In this work, ALP catalyzed the hydrolysis of AAP to AA that could reduce Ag^+^ ions to Ag deposited on the electrode. Although the stripping current peak of Ag on a gold electrode was divisive from gold, it was still difficult to directly measure the Ag signal. Therefore, the deposited Ag was stripped from the gold electrode at 0.7 V and then accumulated on a glassy carbon electrode for ASV measurement.

**Figure 9:**
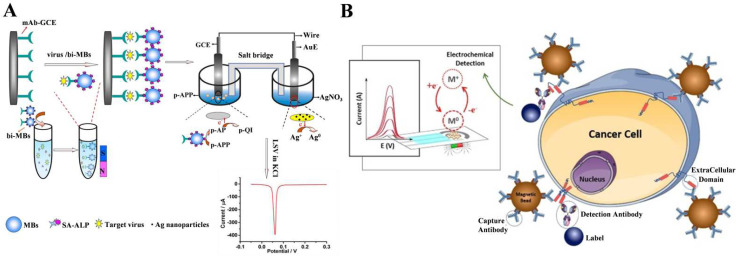
(**A**) Schematic illustration of the protocol for the immunoassay of H7N9 AIV: the target virus was captured by MBs and the sandwich immunoreaction, enzyme-induced metallization reaction mechanism [154]. Copyright 2015 Elsevier. (**B**) Schematic illustration of the electrochemical immunomagnetic assay for the analysis of HER2-ECD based on ALP-induced metallization reaction [161]. Copyright 2020 Elsevier.

Nanomaterials with a large surface area and plenty of functional groups can serve as nanocarriers to load numerous enzymes for signal amplification [144,145,146,147,148]. Qu et al. used ALP-modified silica nanoparticles (SiO_2_ NPs) and ALP-encapsulating liposomes as the signal labels to trigger enzymatic silver metallization for the detection of prostate-specific antigen (PSA) [149,150]. Due to their large surface area and abundant surface functional groups, magnetic nanoparticles (MBs) can serve as carriers to simultaneously load antibodies and a large number of ALP species, increasing the signal-to-noise ratio and improving the sensitivity [151,152,153]. Moreover, thanks to their good magnetic response ability, MBs were popularly utilized as the separation and enrichment supports to capture targets from complex samples. As an example, Wu et al. reported an electrochemical immunosensor for the detection of avian influenza A (H7N9) virus based on immunomagnetic separation and ALP-induced metallization [154]. As shown in Figure 9A, MBs modified with antibodies and ALP were used to capture virus from the samples. After the sandwich immunoreaction, ALP catalyzed the transformation of PAPP to PAP. In the galvanic cell, Ag^+^ ions could be reduced to Ag^0^ by PAP and then deposited on the gold electrode. The dual-electrode signal conversion approach eliminated the potential effect of Ag^+^ ions or silver deposition on enzyme activity. In addition, the combination of 3-indoxyl phosphate (3-IP, the enzymatic substrate) and Ag^+^ allowed the development of versatile ALP-based electrochemical immunosensors for the detection of human anti-gliadin antibodies, cancer antigen 15-3, human epidermal growth factor receptor 2 (HER2-ECD), amyloid-beta 1–42, and so on [155,156,157,158,159,160]. For example, Freitas et al. reported the electrochemical immunomagnetic analysis of HER2-ECD in human serum and cancer cells [161]. As illustrated in Figure 9B, MBs were modified with capture antibody, and the detection antibody was conjugated with ALP. In the presence of HER2-ECD, the sandwich immunecomplex formed on the surface of the MBs that catalyzed the hydrolysis of 3-IP. The products could reduce Ag^+^ ions to Ag deposition on the SPCE surface.

**Figure 10:**
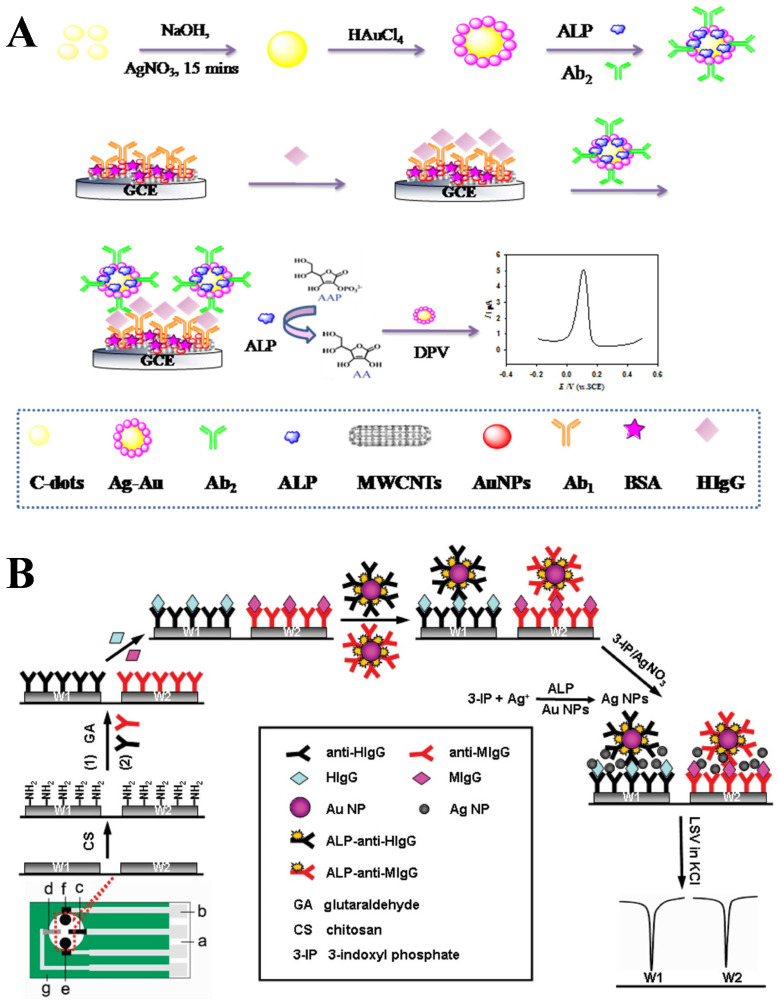
(**A**) Schematic illustration of the electrochemical immunosensor for human IgG detection based on ALP-triggered silver deposition and Ag–Au bimetallic NPs as the catalyst [170]. Copyright 2017 Elsevier. (**B**) Schematic illustration of preparation of immunosensor array and detection strategy by sandwich-type immunoassay and linear sweep voltammetric stripping analysis of enzymatically deposited silver nanoparticles (AgNPs) [171]. Copyright 2011 American Chemical Society.

The accumulation of metal silver on the electrode may block the diffusing of enzymatic products into a solution. Thus, different nanomaterials such as AuNPs, carbon nanotubes (CNTs), and platinum nanoparticles (PtNPs) were used for Ag deposition [162,163,164,165,166]. In an ALP-linked AuNPs-based immunoassay, AuNPs can serve as the nanocarriers for ALP loading and the supports, as well as catalysts for Ag deposition [167,168,169]. Zhang et al. reported an electrochemical immunosensor for human IgG detection based on ALP-triggered Ag deposition and Ag–Au bimetallic nanoparticles as the catalysts (Figure 10A) [170]. In this study, the Ag–Au bimetallic nanoparticles were synthesized with carbon dots (CDs) as the reductant and stabilizer and were further modified with ALP and a detection antibody. After the immunoreaction, the ALP on the electrode catalyzed the hydrolysis of AAP to generate AA, which could induce Ag deposition on the surface of Ag–Au bimetallic nanoparticles. Lai et al. reported a multiplexed immunoassay by integration of ALP-functionalized AuNPs with Ag deposition [171]. As illustrated in Figure 10B, capture antibodies were covalently immobilized on the surface of chitosan-modified SPCE. After the sandwich-type immunoreaction, ALP attached on the SPCE catalyzed the hydrolysis of 3-IP, which could reduce Ag^+^ ions to Ag. Both ALP and AuNPs promoted the deposition of Ag, amplifying the detection signal. This multiplexed immunosensor exhibited a wide linear range over four orders of magnitude for human and mouse IgG detection. To achieve the best performance, the authors further investigated the influence of AuNPs morphology on the detection performance and found that the protocol with irregular-shaped AuNPs showed a better analytical result than that with spherical AuNPs [172].

**Figure 11:**
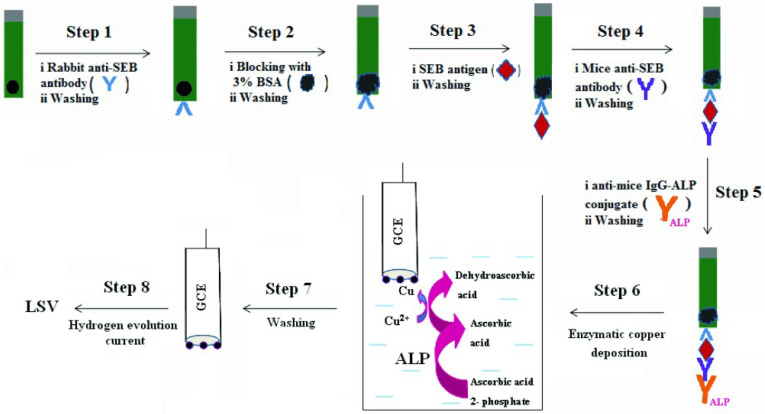
Schematic illustration of the electrochemical immunosensor for SEB detection based on HER inhibition by ALP-catalyzed copper deposition on PtNPs-modified GCE [174]. Copyright 2014 Wiley-VCH.

PtNPs modified on the electrode can generate a strong electrocatalytic current through the hydrogen evolution reaction (HER). AA-induced copper deposition on the PtNPs-modified electrode can lead to a negative shift of the hydrogen evolution potential by the catalytic poisoning of PtNPs. Thus, the ALP-catalyzed generation of AA can be combined with copper deposition and HER for the construction of electrochemical immunosensors [173]. As proof, Sharma et al. developed an electrochemical immunosensor for *Staphylococcal Enterotoxin* B (SEB) detection based on HER inhibition by ALP-catalyzed copper deposition on PtNPs-modified GCE (Figure 11) [174]. After the sandwich immunoreaction, ALP catalyzed the hydrolysis of AAP to AA. Then, the PtNPs-modified electrode was immersed in the resulting solution containing ALP-catalyzed product AA and Cu^2+^ ions for copper deposition. The potential shift value exhibited a linear relationship with SEB concentration in the range of 1 ng/mL–1 μg/mL, with a detection limit of 1 ng/mL.

**Table 1:** Analytical performance of ALP-based electrochemical biosensors.

Detection Principle	ALP Substrate	Target	Linear Range	LOD	Ref.
Direct detection of enzymatic products	PAPP	Digoxin	0.5–2.0 ng/mL	30 pg/mL	[38]
	PAPP	Mouse IgG	10–1000 ng/mL	10 ng/mL	[39]
	PAPP	hCG	0.8–40 units/L	0.8 units/L	[40]
	PAPP	TBG and cortisol	31–1000 μg/L and 1 × 10^2^–2000 nM	NA	[41]
	NPP	PCBs	1 × 10^−5^–1 U/mL	2.1 × 10^−6^ U/mL	[63]
	PNPP	5-methylcytosine	0.01–50 nM	3.2 pM	[70]
	3-IP	*Escherichia coli* O157:H7	6 × 10^4^–6 × 10^7^ cells/mL	6 × 10^3^ cells/mL	[89]
	AAP	Mouse IgG	1–1000 ng/mL	0.3 ng/mL	[99]
	PNPP	hCG	1–100 mIU/mL	0.36 mIU/mL	[109]
ALP catalysis plus EC redox cycling	AAP	Troponin I	100 fg/mL–1 μg/mL	10 fg/mL	[118]
	1A2N-P	CK-MB	100 fg/mL–1 μg/mL	80 fg/mL	[119]
	AAP	APE-1	10 fg/mL–100 pg/mL	3.9 fg/mL	[131]
ALP catalysis plus ECC redox cycling	PAPP	Mouse IgG	0.1–1 × 10^5^ pg/mL	100 fg/mL	[133]
	PAPP	Mouse IgG	1 pg/mL–1 μg/mL	1 pg/mL	[134]
	PAPP	Troponin I	10 fg/mL–1 μg/mL	1 fg/mL	[135]
ALP-based EE redox cycling	PAPP	CEA	5 pg/mL–50 ng/mL	2 pg/mL	[137]
	PAPP	Human IgG	10 fg/mL–1 μg/mL	3.5 fg/mL	[138]
ALP-catalyzed metal deposition	AAP	Human IgG	0.1–50 ng/mL	0.03 ng/mL	[142]
	AAP	Human IgG	5–1000 ng/mL	2.2 ng/mL	[143]
	PAPP	H7N9 virus	0.01–20 ng/mL	6.8 pg/mL	[154]
	3-IP	HER2	5–50 ng/mL	2.8 ng/mL	[161]
	AAP	Human IgG	0.005–100 ng/mL	0.9 pg/mL	[170]
	3-IP	Human and mouse IgG	0.01–250 ng/mL	4.8 pg/mL	[171]
	AAP	Human IgG	10 pg/mL–1 μg/mL	2 pg/mL	[173]
	AAP	SEB	1 ng/mL–1 μg/mL	1 ng/mL	[174]

Abbreviations: PAPP, *p*-aminophenyl phosphate; IgG, immunoglobulin G; hCG, humanchorionic gonadotropin; TBG, thyroxine-binding globulin; NPP, α-naphthyl phosphate; PCBs, polychlorinated biphenyls; PNPP, *p*-nitrophenyl phosphate; AAP, 2-phospho-L-ascorbic acid; 1A2N-P, 1-amino-2-naphthyl phosphate; APE-1, apurnic/apyrimidinicendonuclease; 3-IP, 5-bromo-4-chloro-3-indolyl phosphate; CEA, carcinoembryonic antigen; SEB, *Staphylococcal Enterotoxin* B; HER2, human epidermal growth factor receptor 2.

## 3. PEC Methods

PEC techniques are widely used in various analytical applications due to their impressive advantages in terms of high sensitivity, a simple instrument, and easy operation [175]. The PEC process mainly involves photo-to-electric conversion on a photoelectrode under the illustration of an applied light. The PEC sensing interface and sensing strategy play a major role in the construction of highly efficient PEC biosensors [176]. In recent years, the combination of ALP-based immunoassays and PEC techniques has sparked significant excitement. The electroactive or reducing species produced by ALP catalysis can modulate PEC signals through different mechanisms, including ALP-catalyzed products as electron donors, ALP-mediated redox cycling, and ALP-mediated in situ growth or the bioetching of the photoelectrode (Table 2) [177,178].

### 3.1. ALP-Catalyzed Products as Electron Donors

**Figure 12:**
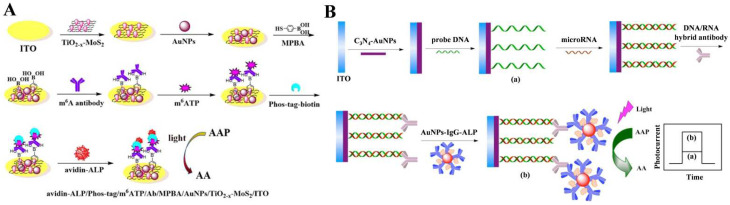
(**A**) Schematic illustration of the preparation process of TiO_2−x_-MoS_2_ and the construction process of the biosensor [188]. Copyright 2021 Elsevier. (**B**) Schematic illustration of the PEC immunosensor for microRNA detection based on IgG–ALP-modified AuNPs. (**a**) DNA/g-C_3_N_4_-AuNPs/ITO; (**b**) ALP/antibody/RNA-DNA/g-C_3_N_4_-AuNPs/ITO [192]. Copyright 2016 Elsevier.

The photoelectrode can be coupled with enzyme catalysis through the interactions between electrons/holes and enzymatic products (oxidative/reductive species). The exciting and transfer of electrons can be promoted or hindered, and the enzymatic reaction-modulated change in the generated photocurrent indirectly reflects the concentration of analytes. ALP can be integrated into PEC immunoassays as an enzyme unit for signal amplification [179,180]. Generally, enzymatic products such as AA could be in situ produced to act as hole-trapping reagents for the capture of the photogenerated holes (h^+^) of photoactive materials, providing electrons to hamper the photogenerated electron–hole recombination and resulting in an increase in the photocurrent signal [181,182,183,184,185]. For example, Yang et al. developed a signal-on PEC immunosensor for the detection of *M.SssI* methyltransfease activity based on the ALP-catalyzed in situ production of AA as an electron donor [186]. Zhang et al. reported a simultaneous PEC immunosensor for dual-cardiac marker detection using ALP and acetylcholineesterase (AChE) as the enzyme tags [187]. Ai et al. developed a PEC biosensor for N^6^-methyladenosine (m^6^A) detection based on ALP catalysis [188]. As presented in Figure 12A, black titanium dioxide (TiO_2−x_) and a molybdenum sulfide (MoS_2_) heterojunction (TiO_2−x_-MoS_2_) were used as the photoactive materials and were further modified with an m^6^A antibody through the interaction between the boronic acid group in *p*-mercaptophenylboronic acid (MPBA) and the glycosyl group in the antibody. After the capture of m^6^ATP by the immunoreaction, Phos-tag-biotin was added to specifically label the phosphate group of m^6^ATP, allowing for the immobilization of avidin-ALP. The attached ALP could catalyze the conversion of AAP toAA by serving as the electron donor to inhibit the recombination of the photogenerated electron and hole of the photoactive material, greatly improving the PEC response. Nevertheless, the 1:1 ration of the target and signal amplification unit (ALP) limited the sensitivity of the method. To improve the ratio of the target and enzyme label, different nanomaterials were used as the carriers to load recognition elements and enzymes [189,190,191]. For instance, Yin et al. developed a PEC immunosensor for microRNA detection using IgG–ALP-modified AuNPs as multi-enzyme labels [192]. As illustrated in Figure 12B, DNA capture probes were immobilized on the AuNPs-g-C_3_N_4_-decorated ITO surface. After hybridization between the capture probe and the target microRNA, the DNA:RNA hybrids were labeled with an anti-DNA:RNA antibody. Then, the IgG–ALP conjugates attached on AuNPs were captured through the specific interaction between the antibody and IgG. ALP immobilized on the electrode catalyzed the hydrolysis of AAP to AA as the electron donor to produce a strong photocurrent. However, the binding of biomolecules (e.g., antibodies, antigens, and enzymes) and nanomaterials on the photoelectrode may decrease the PEC signal because of the steric hindrance effect to mass transfer and the interference in the light harvest of the photoactive substrate [193]. Furthermore, the oxidation capacity of the photovoltaic substrate and light radiation in the PEC system may damage the structure of biomolecules.

**Figure 13:**
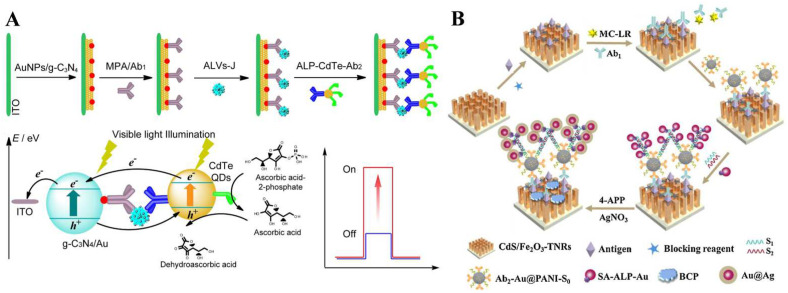
(**A**) Schematic illustration of a dual signal-on PEC immunosensor for detection of ALV-J based on AuNPs/g-C_3_N_4_ coupling with CdTe QDs and in situ enzymatic generation of electron donor [194]. Copyright 2016 Elsevier. (**B**) Schematic illustration of the PEC immunoassay for detection of MC-LR based on HCR-assisted EPI effect and enzymatic biocatalytic precipitation [197]. Copyright 2018 Elsevier.

It is an effective “signal-on” strategy to modify photoactive nanoparticles with detection antibodies and enzymes. After the immune-recognition event, the signal labels can significantly enhance the PEC responses through the synergistic interaction of enzymatic catalysis and photoactive species. Sun et al. developed a dual “signal-on” PEC immunosensor for the detection of subgroup J avian leucosis viruses (ALV-J) based on the integration of AuNPs/g-C_3_N_4_ and CdTe QDs as well as the in situ enzymatic generation of electron donors [194]. As illustrated in Figure 13A, AuNPs/g-C_3_N_4_ as the photoactive electrode materials were modified with capture antibodies. CdTe QDs with light-harvesting ability were functionalized with a detection antibody and ALP. After the immunoreaction, the photocurrent was enhanced due to the matched energy level between the CdTe QDs and AuNPs/g-C_3_N_4_. Meanwhile, the ALP-catalyzed in situ produced AA further enhanced the photocurrent response, realizing the dual “signal-on” mode for the PEC assay.

Coupling the enzymatic reaction or plasmonic metal nanoparticles with the steric hindrance effect is also an effective strategy to reduce the PEC response through multiple amplification strategies. For example, Zhang et al. reported the PEC and visualized immunoassay of β-human chorionic gonadotrophin based on enzymatic biocatalytic precipitation [195]. In this work, ALP catalyzed the oxidative hydrolysis of 5-bromo-4-chloro-3-indoyl phosphate (BCIP) to form indigo precipitates. The in situ performed insulating layer reduced the photocurrent by impeding the interfacial mass and electron transfer. The exciton–plasmon interaction (EPI) between photoactive CdS QDs and plasmonic Ag/Au NPs caused significant photocurrent attenuation through the energy transfer effect. The CdS QDs- and AgNPs/AuNPs-based system was well-exploited for PEC analysis [196]. Wei et al. reported a PEC immunosensor for the detection of microcystin-LR (MC-LR) based on the hybridization chain reaction (HCR)-assisted EPI effect and enzymatic precipitation [197]. As displayed in Figure 13B, the sandwich immunoreaction occurred on the surface of CdS/Fe_2_O_3_ co-sensitized TiO_2_NR arrays/the ITO electrode. After the HCR reaction on DNA-primer-modified Au@polyaniline nanocomposites, the resulting DNA polymers with multiple biotin labels could capture an increasing number of SA-ALP-modified AuNPs via specific biotin–SA interactions. Then, ALP catalyzed the transformation of PAPP to PAP. In the presence of Ag^+^ ions, PAP induced the Ag deposition reaction on the photoelectrode to generate Au@Ag for the PEI effect and an insoluble biocatalytic precipitation (BCP) of benzoquinone serving as an insulating layer and an electron acceptor to inhibit the electron transfer between the solid–liquid interface.

**Figure 14:**
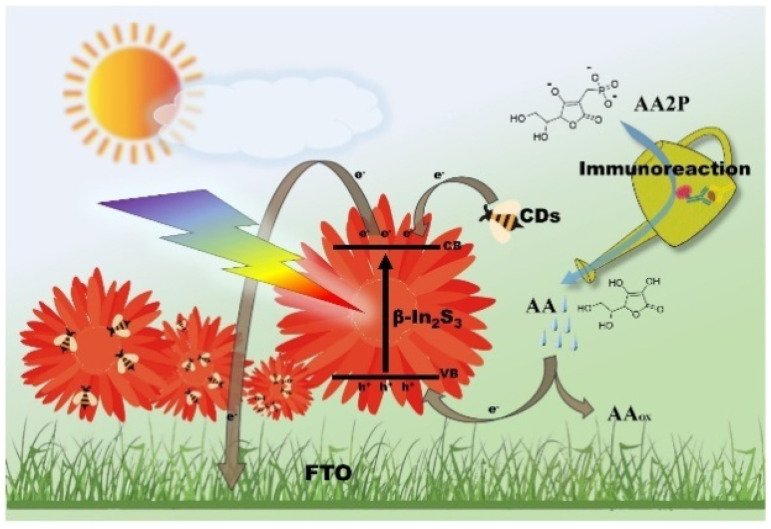
Schematic illustration of the PEC immunosensing system for the detection of AFP on β-In_2_S_3_@CDs photoelectrode by coupling with enzyme immunoassay format [199]. Copyright 2017 Elsevier.

To avoid the steric hindrance effect and the potential damage of biomolecules, the split-type detection mode was widely adopted for various PEC immunoassays, in which the immunoreaction process was separated from the PEC detection system [198]. The utilized optical spectrum region of most photoactive materials is always in the limited region like ultraviolet (UV) and visible (Vis) light. To take advantage of the overall luminous energy, Yu et al. developed a full-spectrum-responsive PEC immunosensor for the detection of alpha-fetoprotein (AFP) based on β-In_2_S_3_@CDs nanoflowers [199]. As illustrated in Figure 14, the flower-like β-In_2_S_3_@CDs hybrid materials prepared via a one-pot hydrothermal method could enhance the photocurrent signal under UV, Vis, and near-infrared (NIR) irradiation. AA generated in the ALP-mediated immunoreaction served as a photoanode sacrificial agent to reduce the electron–hole pair recombination, increasing the PEC signal in the glare of axenon lamp.

**Figure 15:**
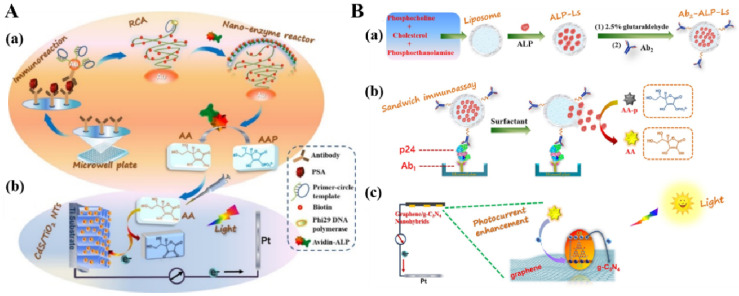
(**A**) Schematic illustration of (**a**) immunoreaction-induced ALP-mediated nanoenzyme reactor formation through RCA, and (**b**) enzymatic product AA-mediated hole-trapping in CdS QD-sensitized TiO_2_nanotube array for the amplification of PEC response [201]. Copyright 2015 American Chemical Society. (**B**) Schematic illustration of (**a**) preparation process of Ab_2_–ALP–Ls signal label, (**b**) sandwich immunoassay based on Ab_2_–ALP–Ls signal label coupling with ALP-catalyzed generation of AA, and (**c**) amplification of photocurrent signal based on AA-mediated hole-trapping in GR/g-C_3_N_4_ electrode [207]. Copyright 2017 Elsevier.

DNA-based amplification techniques are considered as powerful tools to amplify the signals of different biosensors [200]. Such techniques can also be introduced into PEC immunoassays for signal enhancement. Zhuang et al. developed a split-type PEC immunosensor for PSA detection by combining the RCA reaction with the ALP-triggered in situ electron donor-producing strategy [201]. As presented in Figure 15A, the immunoreaction was conducted on a microplate with secondary antibody/primer-circular DNA-labeled AuNPs as the detection tags. After the RCA reaction, a large number of the repeated biotin-functionalized DNA sequences were in situ generated on AuNPs to capture a large number of avidin–ALP conjugates. Next, the resulting solution containing enzymatic products (AA) was transferred to the PEC cell, greatly quenching the photogenerated holes in the CdS QDs-sensitized TiO_2_ nanotube arrays. However, DNA-based PEC immunosensors are always limited by the time-consuming reactions and the use of extra enzyme-conjugated labels.

Liposome composed of phospholipid bilayers with hollow cavity can carry various guest species for biosensing applications, such as small molecules, enzymes, and nanomaterials [202,203,204,205,206]. They were also used in ALP-linked liposomal PEC immunoassays for multiple signal amplification. For example, Zhuang et al. reported a split-type ALP-encapsulated liposomal PEC immunoassay for HIV-p24 antigen (p24) detection [207]. As displayed in Figure 15B, liposome (Ls) was loaded with ALP in its aqueous cavity and then modified with detection antibody Ab_2_ to form an Ab_2_–ALP–Ls signal label. After the immunoreaction in the plate, ALP molecules released from Ls under the treatment with Tween 20 catalyzed the hydrolysis of AAP to AA. When the resulting solution was added to the PEC detection cell, the produced AA restrained the electron–hole recombination in g-C_3_N_4_, increasing the photocurrent signal of the graphene/g-C_3_N_4_ nanohybrids (GR/g-C_3_N_4_)-based photoelectrode.

**Figure 16:**
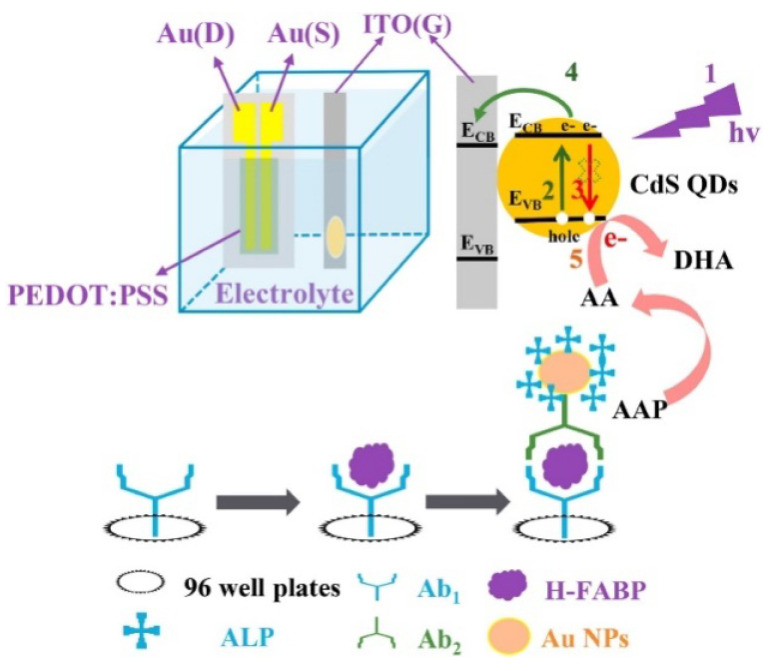
Schematic illustration of the AA-mediated OPECT sensing strategy (process 1: light irradiation, process 2: excitation of valence band electrons, process 3: recombination of photogenerated holes and electrons, process 4: electrons transfer from CdS QDs to ITO electrode, and process 5: scavenge of photogenerated holes by AA) [209]. Copyright 2022 Elsevier.

Recently, the organic PEC transistor (OPECT) technique, by the integration of PEC analysis with an organic electrochemical transistor, showed extraordinarily high sensitivity in the determination of low-abundance analytes. With a photoelectrode as the gate electrode, the enzymatic-reaction-triggered small change in photovoltage can be significantly amplified by the channel current (*I*_DS_) between the source electrode and the drain electrode [208]. For this view, Shi et al. reported an ALP-mediated OPECT sensing strategy for the detection of the heart-type fatty acid binding protein (H-FABP) [209]. Referring to Figure 16, the primary antibody/H-FABP/secondary antibody–AuNPs–ALP sandwich immunecomplexes were produced in a 96-well plate via the specific immunoreaction. Then, the reaction solution was transferred to the OPECT cell, and the enzymatic product AA, serving as a sacrificial reagent, scavenged the photogenerated hole on the valence band (VB) of CdS QDs, leading to a change in the effective gate voltage ($V_{G}^{\mathrm{eff}}$) and *I*_DS_ of the devices. The concentration of H-FABP was sensitively determined by measuring the corresponding *I*_DS_.

**Figure 17:**
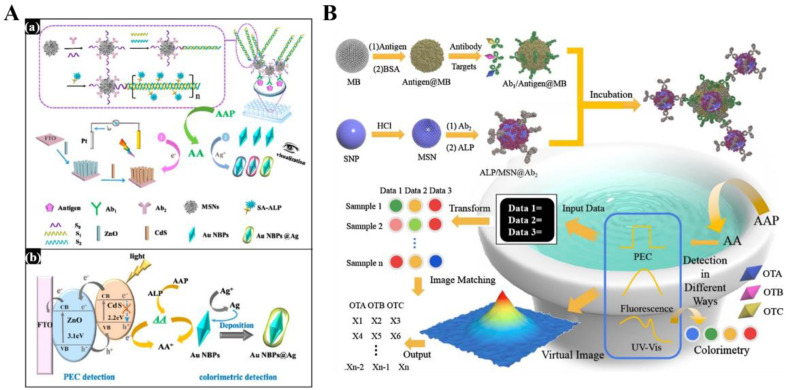
(**A**) Schematic illustration of the construction (**a**) and the response mechanism (**b**) of dual-modal HCR and ALP catalysis-based PEC and colorimetric immunosensor [212]. Copyright 2018 American Chemical Society. (**B**) Schematic illustration of the construction of the immunosensor for multiple ochratoxins; the signal generation of PEC, fluorescence, and colorimetry; the signal transformation; and machine learning [213]. Copyright 2021 Elsevier.

Most PEC immunosensors were designed based on the individual signal change caused by the recognition event, in which the response may be influenced by external interferences, such as operating personnel and different experimental environments. As a result, the dual-signal detection mode was widely adopted for PEC immunoassays to improve the accuracy and sensitivity [210,211]. Wei et al. constructed a dual-modal split-type PEC immunosensor for the detection of MC-LR based on HCR and ALP catalysis [212]. As showed in Figure 17A, the immunoreaction was conducted in amicroplate well, and mesoporous silica nanospheres were employed as the carriers to simultaneously load Ab_2_ and DNA primers to initiate the HCR reaction. The formed biotin-suspended DNA polymers could capture many ALP molecules to catalyze the hydrolysis of AAP. The generated AA could quench the holes generated from CdS/ZnO hollow nanorod arrays (HNRs), achieving a “signal-on” PEC assay. Meanwhile, AA serving as a reducing reagent promoted the in situ formation of silver shells on Au nanobipyramids (Au NBPs), resulting in a series of vivid color variations and blue shifts of the localized surface plasmon resonance (LSPR) band. Recently, machine learning was combined with different analytical methods to develop novel biosensors. Qileng et al. presented imaging-matching-based machine learning for the development of a three-mode broad-specificity immunosensor for the detection of multiple ochratoxins, including ochratoxin A (OTA), ochratoxin B, and ochratoxin C [213]. As shown in Figure 17B, after the enzymatic hydrolysis of AAP during the immunoreaction, the generated AA was used to induce PEC, fluorescence, and the colorimetric reaction. In this method, AA serving as a reducing agent could quench the photogenerated hole and enhance the photocurrent of CdS QDs, inducing the Ag metallization of AuNPs with a color change from blue to red and reducing Ce^4+^ to Ce^3+^ with an intense fluorescence at 360 nm.

### 3.2. ALP-Mediated Redox Cycling

**Figure 18:**
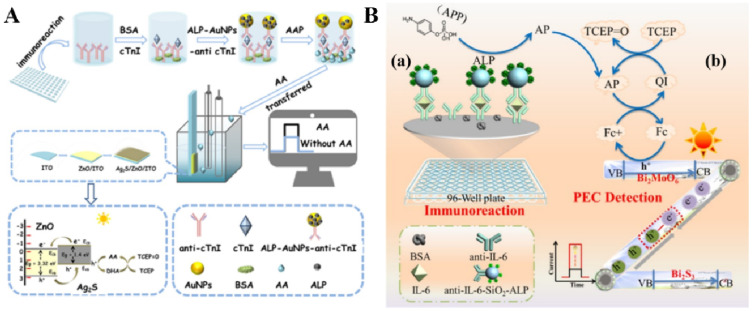
(**A**) Schematic illustration of the split-type chemical redox-cycling-amplification-based PEC immunosensor for detection of cTnI [218]. Copyright 2021 Elsevier. (**B**) Schematic illustration of the PEC bioanalysis (**a**) for IL-6 based on PECCC redox cycling amplification (**b**) [219]. Copyright 2021 American Chemical Society.

As mentioned above, redox-cycling-based amplification can be perfectly combined with an enzymatic reaction to repeatedly regenerate the consumed catalytic products based on well-coupled oxidation–reduction reactions. Given this concept, Cao and co-workers constructed a series of novel PEC platforms based on the fusion of redox cycling amplification and an appropriate photoelectrode for the detection of myoglobin and interleukin-6 (IL-6) [214,215,216,217]. A typical example is the detection of cTnI based on photogenerated hole-induced chemical redox cycling amplification [218]. As shown in Figure 18A, during the immunoreaction in a 96-well plate, ALP attached on AuNPs catalyzed the hydrolysis of AAP tothe signal-reporting species AA. Subsequently, AA served as the electron donor to quench the photogenerated holes of Ag_2_S/ZnO nanocomposites. The oxidation product (dehydroascorbic acid, DHA) at the electrode was repeatedly reduced to AA under the TCEP-mediated chemical redox cycling reaction, eventually leading to an enhanced PEC response. Comparatively, photogenerated hole-induced chemical−chemical (PECCC) redox cycling amplification involving the signaling species recycled by two different reducing (or oxidizing) agents and an appropriate photoelectrode can lead to much faster redox reactions and the regeneration of signaling species. Cao et al. reported a PEC method for IL-6 detection based on PECCC redox cycling for advanced signal amplification [219]. As shown in Figure 18B, the oxidation of Fc by the holes in the Z-scheme Bi_2_S_3_/Bi_2_MoO_6_ heterostructure photoelectrode under illumination triggered the PECCC redox cycling amplification system among the redox mediator Fc, the ALP-participated enzymatic generation of signaling unit AA, and the reducing agent TCEP. Under triple signal amplification, the proposed method exhibited a very low detection limit (2 × 10^−14^ g/mL)and a wide linear range (5 × 10^−14^ to 1 × 10^−8^ g/mL).

### 3.3. ALP-Mediated In Situ Growth or Bioetching of Photoelectrode

**Figure 19:**
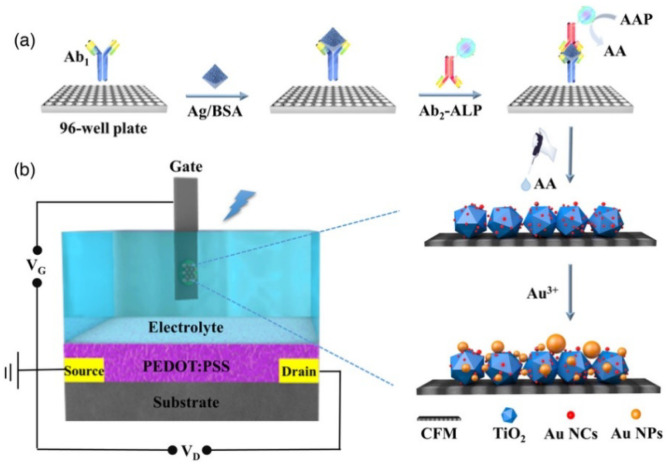
Schematic illustration of (**a**) the sandwich immunorecognition with ALP labels to catalyze the growth of Au NCs toAu NPs in 96-well plate and (**b**) the operation mechanism of the OPECT biosensor with a bio-regulated gate photoanode [221]. Copyright 2021 Wiley-VCH.

Apart from serving as electrode substrates or signal labels in sandwich assays, photoactive materials can be in situ grown on the surface of a photoelectrode to regulate PEC signals. The ALP-mediated in situ enzymatic growth of photoactive materials was popularly combined with PEC techniques for signal amplification. ALP-catalyzed reductive products, AA and IP, can reduce Au^3+^ and Ag^+^ ions to AuNPs and AgNPs on the photoelectrode, leading to a change in PEC signals [220]. Lu et al. developed an OPECT immunosensor for the detection of C-reactive protein (CRP) based on the ALP-mediated regulation of a light-sensitive gate electrode [221]. As displayed in Figure 19, ALP-conjugated mAb_2_ was used in the sandwich immunoassay. AuNCs as the photosensitizers were immobilized on TiO_2_ supported by a 3D carbon fiber matrix (CFM) to improve photon-to-electron conversion efficiency. The enzymatic product AA could reduce Au^3+^ions to AuNCs as the crystalline seeds to promote the formation of plasmonic AuNPs. The light source used was unable to effectively trigger the SPR effect, thereby decreasing the photon-to-electron conversion efficiency and weakening the PEC signal [222].

**Figure 20:**
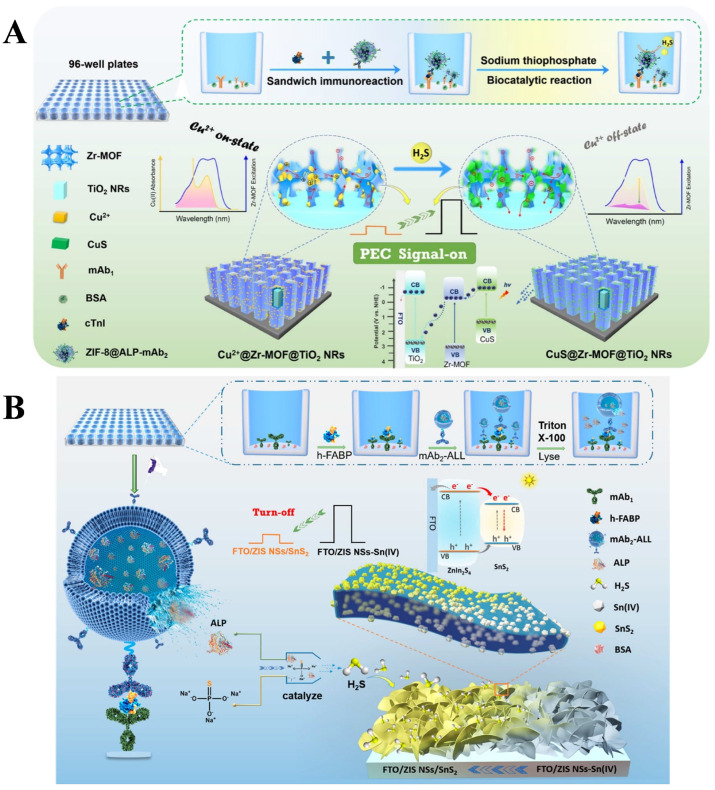
(**A**) Schematic illustration of the signal-on PEC immunoassay based on the tunable competitive absorption of Cu(II) ions onto a MOF-based heterojunction [226]. Copyright 2022 American Chemical Society. (**B**) Schematic illustration of the ALP-loaded liposome-mediated PEC immunoassay based on the in situ formation of type-I heterojunction on an FTO electrode [227]. Copyright 2022 American Chemical Society.

ALP can catalyze the decomposition of sodium thiophosphate (Na_3_SPO_3_, TP) into orthophosphate (PO_4_^3−^) and H_2_S. The resulting H_2_S can react with metal ions or active molecules to in situ prepare materials that can change the PEC signal [223]. PEC immunosensors based on the ALP-mediated enzymatic in situ generation of QDs were developed for the detection of antibody and human serum albumin, in which the produced H_2_S interacted with Cd^2+^ to form CdS QDs that could be determined by the PEC technique [224,225]. Gao et al. developed a tunable competitive absorption-induced “signal-on” PEC immunosensor for cTnI detection based on the Zr-scheme MOF heterojunction and the enzyme-triggered growth of photoactive materials [226]. As shown in Figure 20A, a Zr-MOFs@TiO_2_ nanorods (NRs) electrode exhibiting a high photoelectric response was synthesized by a solvothermal method. The electrode modified with Cu(II) by an electrostatic interaction quenched the PEC signal. Zeolitic imidazolate framework-8 nanoparticles (ZIF-8 NMOFs) were loaded with ALP and mAb_2_ to form ZIF-8@ALP–mAb_2_ complexes. After the formation of the sandwich immunocomplexes in 96-well plates, the enzymatic product H_2_S competitively reacted with Cu(II) to quickly form CuS with a high negative potential of the conduction band (CB). The formed charge-carrier migration pathway resulted in the enhancement of the PEC signal. However, the type-I heterojunction was not a suitable candidate for PEC biosensing due to the unmatched CB and valence band (VB) levels of semiconductors A and B. The in situ combination or growth on a photoelectrode can endow a target-dependent type-I heterojunction with more possibilities in PEC bioassays. Gao et al. reported a liposome-aided type-I heterojunction growth method for a PEC immunoassay of h-FABP [227]. As illustrated in Figure 20B, a fluorine-doped tin oxide (FTO)/ZnInS nanosheets (ZIS NSs)-Sn(IV) electrode was fabricated as the working electrode. ALP-loaded liposome was used as the signal label in the sandwich immunoassay. Under the lysis treatment, the released ALP could catalyze the production of H_2_S to immediately react with Sn(IV) for the in situ formation of the ZIS NSs/SnS_2_ type-I heterojunction on the FTO/ZIS NSs-Sn(IV) electrode. This changed the photogenerated electron−hole transfer path of the photoelectrode, leading to a reduction in the current intensity.

**Figure 21:**
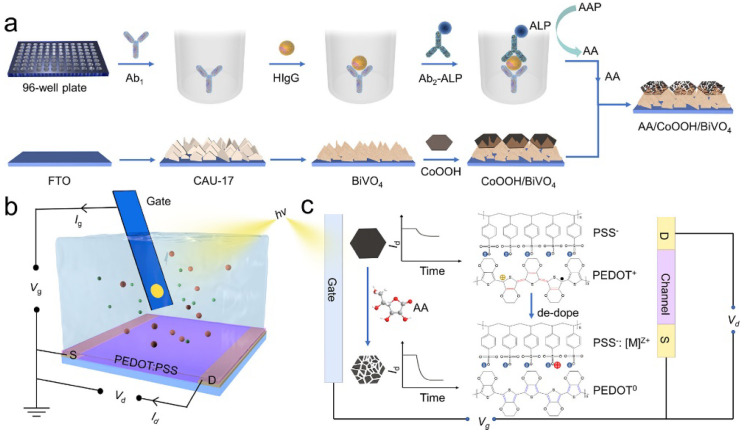
Schematic illustration of (**a**) ALP-labeled sandwich immunocomplexing in a 96-well plate to produce AA for bioetching of the as-fabricated CoOOH/BiVO_4_, (**b**) the OPECT configuration, and (**c**) the corresponding modulation mechanism [231]. Copyright 2023 American Chemical Society.

The enzymatic-bioetching-mediated photocurrent change/shift is a useful approach for PEC biosensing via the controllable dissociation of photoactive materials and the manipulation of the light-harvesting gates [204,228]. Cobalt oxyhydroxide (CoOOH) NSs exhibit an excellent light absorption capacity. They can be easily decomposed by the enzymatic product AA. CoOOH NSs coated on the electrode surface can block the electrolyte contact and light accessibility to the photoelectrode, leading to a decrease in the PEC signal. ALP-catalyzed product AA can etch CoOOH NSs and restore the PEC signal [229]. Zhang et al. developed a PEC biosensor for carcinoembryonic antigen (CEA) detection by coupling HCR with the ALP-mediated bioetching of CoOOH NSs [230]. Ban et al. reported a “signal-on” OPECT immunosensor for human IgG detection based on the ALP-mediated bioetching of the CoOOH/BiVO_4_ gate [231]. As shown in Figure 21, the FTO electrode was gradually covered with CAU-17 MOF-derived BiVO_4_ and CoOOH NSs, resulting in a PEC “signal-off” mode. After the completion of the ALP-linked sandwich immunoassay, the catalytic product AA was collected and then dropped onto the CoOOH/BiVO_4_-modified gate electrode. CoOOH NSs were etched by AA, and BiVO_4_ was partially exposed, resulting in the recovery of the PEC signal. The current change during the ALP-mediated bioetching process was monitored by the polymeric poly(3,4-ethylenedioxythiophene):poly(styrene sulfonate) (PEDOT:PSS) channel.

MnO_2_ NSs, with a band gap of about 2.1 eV and an absorption peak at around 380 nm near the visible regime, can be reduced to Mn^2+^ ions by the enzymatic product, such as H_2_O_2_ and AA. This mechanism was successfully used to develop MnO_2_ NSs-based biosensors [232,233]. For example, Lin et al. developed a PEC immunosensor for AFP detection based on the ALP-mediated bioetching of photoactive carbon quantum dots (CQDs)-functionalized MnO_2_ NSs [234]. In this work, the ALP-triggered dissolution of MnO_2_ NSs led to the release of CQDs from the electrode, followed by a decrease in the PEC current.

**Table 2:** Analytical performance of ALP-based PEC biosensors.

Detection Principle	ALP Substrates	Target	Linear Range	LOD	Ref.
ALP-catalyzed products as electron donors	AAP	*M.SssI*MTs	1–50 unit/mL	0.33 unit/mL	[186]
	AAP	N^6^-methyladenosine	0.005–35 nM	2.57 pM	[188]
	AAP	MiRNA	5–3000 fM	2.26 fM	[192]
	AAP	AFP	0.5–50 ng/mL	37.9 pg/mL	[199]
	AAP	PSA	0.001–3 ng/mL	0.32 pg/mL	[201]
	AAP	HIV-p24 antigen	1 pg/mL–50 ng/mL	0.63 pg/mL	[207]
	AAP	Microcystin-LR	0.05 ng/mL–5 μg/mL	0.03 pg/mL	[212]
ALP-mediated redox cycling	AAP	Myoglobin	1 × 10^−4^–100 ng/mL	0.1 pg/mL	[214]
	AAP	Myoglobin	1 × 10^−4^–100 ng/mL	0.03 pg/mL	[215]
	AAP	Troponin I	10 fg/mL–1 ng/mL	3 fg/mL	[218]
	PAPP	Interleukin-6	50 fg/mL–10 ng/mL	20 fg/mL	[219]
ALP-mediated in situ growth or bioetching of photoelectrode	AAP	CRP	1 pg/mL–200 ng/mL	0.1 pg/mL	[221]
	AAP	h-FABP	0.5 pg/mL–50 ng/mL	0.1 pg/mL	[222]
	thiophosphate	Troponin I	0.01–10 ng/mL	8.6 pg/mL	[226]
	thiophosphate	h-FABP	0.1–1000 pg/mL	55 fg/mL	[227]
	AAP	Aflatoxin B_1_	0.01–10 ng/mL	2.6 pg/mL	[229]
	AAP	CEA	0.01–100 ng/mL	5.2 pg/mL	[230]
	AAP	Human IgG	1 × 10^−4^–100 ng/mL	25 fg/mL	[231]
	AAP	Alpha-fetoprotein	0.01–100 ng/mL	9.3 pg/mL	[234]

Abbreviations: MTs, methyltransfease; AFP, alpha-fetoprotein; PSA, prostate specific antigen; h-FABP, heart-type fatty acid binding protein; AAP, 2-phospho-L-ascorbic acid; CEA, carcinoembryonic antigen.

## 4. ECL Methods

**Figure 22:**
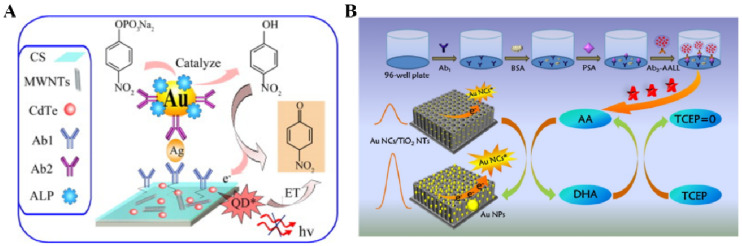
(**A**) Schematic illustration ofthe principle for amplified energy transfer ECL quenching for sensitive detection of CEA based on the ALP-catalyzed in situ generation of molecular quenchers [240]. Copyright 2013 Elsevier. (**B**) Schematic illustration of the ECL immunoassay for PSA detection based on liposome, ALP catalysis, chemical redox cycling, and in situ growth of AuNPs [241]. Copyright 2022 Elsevier.

ECL is a type of luminescence produced from the combination of an electrochemical reaction and a chemiluminescence (CL) reaction. The technique possesses the advantages of both electrochemical and CL methods. During the ECL process, the electrochemically produced species at/near the electrode surface can react with each other to form the excited state of luminophore-related compounds, thus lighting up the luminescence. Unlike electrochemical biosensors, ECL assays are scarcely influenced by the background current and the potential window of the electrode. Thus, the ECL technique was successfully integrated with enzyme-linked immunoassays [235,236,237,238,239]. As the key components of ECL sensing platforms, various nanomaterials with good chemical stability and excellent signals were used as luminophores to construct various immunosensors. Like the Förster resonance energy transfer (FRET) mechanism in fluorescence biosensors, the excited-state nanomaterials can be quenched through a charge transfer or energy transfer. Given this concept, Yang et al. reported an ECL immunosensor for the detection of CEA based on the ALP-catalyzed in situ generation of molecular quenchers [240]. As shown in Figure 22A, a mixture of chitosan-multiwalled CNTs (MWCNTs) and CdTe QDs was deposited on the electrode. ALP-modified AuNPs were used to increase the number of enzyme labels per immunoreaction event. ALP-catalyzed product PNP was electrochemically oxidized to *p*-benzoquinone (PBQ), which could quench the luminescence of excited CdTe QDs through the energy transfer from QDs to PBQ.

AuNCs as fluorophores can produce an ECL signal and, meanwhile, serve as the seeds for gold metallization. Thus, an ALP-mediated immunoassay can be integrated with a AuNCs-based ECL system to modulate the signal through in situ metallization. Cao et al. proposed a novel strategy by coupling liposome, ALP catalysis, chemical redox cycling, and the in situ growth of AuNPs to develop an ECL immunosensor for PSA detection [241] As shown in Figure 22B, after the immunoreaction, many ALP molecules released from the liposome catalyzed the conversion of AAP to AA. The produced AA promoted the growth of AuNCs to AuNPs in the presence of Au^3+^ ions. In this process, AA was oxidized to DHA that could be immediately reduced back to AA by excess TCEP for the next reduction of Au^3+^. Under chemical redox cycling, the repeated regeneration of AA resulted in the formation of AuNPs and greatly enhanced the ECL intensity.

## 5. Conclusions and Perspectives

In recent years, in parallel with the significant progress in nanotechnology and bioconjugation chemistry, great advances in the development of ALP-based electrochemical immunoassays have been achieved. To increase the sensitivity and reduce the background, significant efforts have been made to integrate various signal-amplified strategies with electrochemical immunoassays. Many novel works and promising results were systematically summarized in this work. In particular, there was a significant achievement in the strategies by combining ALP catalysis with EC and ECC redox cycling for improving sensitivity. The loading of multiple ALP molecules on the nanomaterials with excellent electrocatalytic activity also significantly improved detection performance under the synergistic catalysis. Split-type immunoassays, by separating the immunoreaction from the detection system, can avoid the interference from complex samples and decrease the background signal. Abundant substrate/product pairs and effective ALP catalysis provide more promising ways to perfectly couple electrochemical immunoassays with emerging powerful strategies.

In spite of these advancements, there are still some challenges facing the applications of ALP-based electrochemical immunosensors. First, the requirement of multiple incubation and washing steps may limit the application of ALP-based immunoassays in terms of fast, on-line, and automated analyte detection. In addition, some methods were only used for the assays of samples in buffer solutions but not of real biological matrices. Second, for the preparation of enzyme-modified nanomaterials, existing immobilization methods may face several problems such as enzyme leaching and denaturation, complex processes, and decreased recognition ability. The in situ formation or encapsulation of enzymes on nanocomposites through mild and one-pot methods may be an effective solution to this problem. Third, considering the widespread application of nanomaterials and a nanostructured surface in multienzyme labeling and antibody immobilization, the size, shape, or composition of nanomaterials and the bioconjugation efficiency during the preparation of ALP-modified nanocomposites may affect the reproducibility and accuracy of measurement results. Fourth, although smartphone-based Point-of-Care Testing (POCT) has become a popular research hotspot due to its portability and low cost, most ALP-based immunoassays are limited to the requirements of strict experimental conditions, specialized instruments, and professional personnel, which may hamper the realization of POCT. Thus, greater efforts should be made to integrate ALP-based immunoassays into smartphone-based devices (e.g., lab-on-a-chip, microfluidic analysis, and μPAD).

## Data Availability

Not applicable.
